# Effect of Dopaminergic D1 Receptors on Plasticity Is Dependent of Serotoninergic 5-HT1A Receptors in L5-Pyramidal Neurons of the Prefrontal Cortex

**DOI:** 10.1371/journal.pone.0120286

**Published:** 2015-03-16

**Authors:** Claire Nicole Jeanne Meunier, Jacques Callebert, José-Manuel Cancela, Philippe Fossier

**Affiliations:** 1 Neuroscience Paris-Saclay Institute (NeuroPSI), UMR 8197 CNRS-Université Paris-Sud, Bâtiment 446, Université Paris-Sud, Orsay F-91405, France; 2 Université Paris Descartes, Laboratoire de Neuropharmacologie des addictions, INSERM U705 CNRS UMR 7157, 4 avenue de l’Observatoire, 75006 Paris, France; Centre national de la recherche scientifique, University of Bordeaux, FRANCE

## Abstract

Major depression and schizophrenia are associated with dysfunctions of serotoninergic and dopaminergic systems mainly in the prefrontal cortex (PFC). Both serotonin and dopamine are known to modulate synaptic plasticity. 5-HT_1A_ receptors (5-HT_1A_Rs) and dopaminergic type D1 receptors are highly represented on dendritic spines of layer 5 pyramidal neurons (L5PyNs) in PFC. How these receptors interact to tune plasticity is poorly understood. Here we show that D1-like receptors (D1Rs) activation requires functional 5HT_1A_Rs to facilitate LTP induction at the expense of LTD. Using 129/Sv and 5-HT_1A_R-KO mice, we recorded post-synaptic currents evoked by electrical stimulation in layer 2/3 after activation or inhibition of D1Rs. High frequency stimulation resulted in the induction of LTP, LTD or no plasticity. The D1 agonist markedly enhanced the NMDA current in 129/Sv mice and the percentage of L5PyNs displaying LTP was enhanced whereas LTD was reduced. In 5-HT_1A_R-KO mice, the D1 agonist failed to increase the NMDA current and orientated the plasticity towards L5PyNs displaying LTD, thus revealing a prominent role of 5-HT_1A_Rs in dopamine-induced modulation of plasticity. Our data suggest that in pathological situation where 5-HT_1A_Rs expression varies, dopaminergic treatment used for its ability to increase LTP could turn to be less and less effective.

## Introduction

The prefrontal cortex (PFC) is a critical region of the brain that controls many cognitive functions such as working memory [1], social behavior [2] and decision making [3]. Cortical networks of the PFC are endowed with a complex cellular machinery controlling synaptic plasticity and are therefore capable of modulating the neuronal activity associated with cognitive processes [4]. These modulations of neuronal activity are known to be under the control of various neurotransmitters such as serotonin (5-HT) and dopamine (DA). Dysfunction of these mechanisms are observed in many neurological disorders, including schizophrenia [5], bipolar disorder [5], attention deficit/hyperactivity disorder [6] and Alzheimer’s disease [7].

Synaptic plasticity is often competitive and dependent on the excitation-inhibition balance of neuronal circuits [8,9]. In pyramidal neurons of cerebral cortices, excitatory synaptic inputs can be correlated with glutamate sensitivity and are associated with the induction of the long-term potentiation (LTP) [10] and long-term depression (LTD). Central to glutamate action, the NMDA receptors (NMDAR) activation could lead to either LTP or LTD, depending of the magnitude of the Ca^2+^ entry. In addition, recent advances suggest that GABAergic inhibition could modulate the local dendritic Ca^2+^ transients underlying the induction of plasticity [11].

Both serotoninergic and dopaminergic inputs to the PFC have been shown to interact with the synaptic plasticity [12,13]. In the PFC, 5-HT1A receptors (5-HT1ARs) are highly expressed in pyramidal and GABAergic neurons [14,15]. 5-HT1ARs are generally known for their inhibitory effect on LTP at perforant pathway-dentate synapses in hippocampus *in vitro* [16]. In a previous study, we demonstrated that postsynaptic 5-HT1ARs activation, through a modulation of NMDARs, facilitate LTD in the PFC [17]. In the case of DA, the effects are mediated by the D1-class receptors (D1 and D5) and by the D2-class receptors (D2, D3, D4) [18]. Numerous histological studies in the PFC have confirmed that DARs are strongly expressed with a widespread distribution of D1Rs (review by [19]). Afferent dopaminergic fibers [20] project on D1 receptors localized on the distal dendrites of pyramidal neurons in deep layer V-VI and on GABAergic neurons (non spiny fast spiking neurons) in the PFC [21–23]. Many electrophysiological studies have shown that dopamine D1-like receptors (D1Rs) favors LTP induction at hippocampal-PFC synapses and enhanced NMDA-mediated responses in PFC [24,25]. However, the situation appears to be complex since an “inverted-U” shape relation between the extracellular dopamine level and plasticity induction in the rat PFC has been described [26,27]. For instance, the activation of D1Rs due to an excess level of DA impairs LTD-induction and consequently it has been proposed that the degree of D1Rs stimulation determines the profile of plasticity [28].

The modulation of plasticity is crucial in the modeling of output signals from the PFC to subcortical area and an aberrant plasticity may underlie cognitive deficits in psychiatric disorders [27]. Importantly, it appears that anatomical and pharmacological studies have shown that the serotoninergic system modulates the dopaminergic system [29]. Furthermore, several studies have pointed out that 5-HT modulate DA levels in the PFC possibly through post-synaptic 5-HT1ARs [30]. It has been shown that the number of post-synaptic 5HT1ARs dramatically decreased in humans suffering from major depression [31,32]. However, the precise impact of the interaction between 5-HT1ARs and D1Rs on the synaptic plasticity in PFC is poorly known and represents therefore an important goal.

Our aim was to investigate the role of D1Rs in the orientation of the plasticity of L5PyNs (LTP or LTD) induced by a High Frequency of Stimulation (HFS) in the PFC. In particular, we investigated the potential role of 5HT1A receptors in the modulation of plasticity by D1Rs. Taking advantage of 5-HT1AR-KO mice, we compared the actions of agonist and antagonist of D1Rs on synaptic plasticity. Our main results bring a new step in the understanding of DA effects in the PFC. Here we show that the activation of D1Rs, which favors LTP through a modulation of NMDA receptors, requires functional 5-HT1ARs.

## Methods

### Animals

Paired wild-type 129/Sv and 5-HT1AR-KO mice [33] were bred under standard conditions in our animal facilities (INSERM U894-CPN; CNPS). All efforts were made to reduce the number of animals used. The agreement number for our animal facilities is: C 91–471–104. Our study includes exclusively in vitro experiments with no in vivo work. The initial experiments performed before 2013, were authorized by the Essonne Préfecture (number of agreement: 91–343 de la direction des services vétérinaires). Then, the experiments were done in accordance with the European and Institutional guidelines for the care and use of laboratory animals (Council Directive 86/609/EEC and 2010/63/UE and its application in 2013 by the French National Research Council. The article 3 of the 2010/63/UE directive permits to euthanatize animals by cervical dislocation to excise brain tissues for experiments without any requirement of a specific ethical committee agreement. P21 to P28 mice were killed by cervical dislocation and their brains quickly removed.

Genotyping was carried out by polymerase chain reaction applied to genomic DNA from tail biopsies with appropriate primers. Wild-type (n = 38 animals) and mutant (n = 38 animals) mice were the product of mating between heterozygous couples raised on the same 129/Sv genetic background [34].

### HPLC quantification

Prefrontal cortex from individual mice (P21 to P28) were homogenized in ice-cold 10^−3^ M chlorhydric acid containing sodium metabisulfite (10 μM), EDTA (10 μM) and ascorbic acid (10 μM). After centrifugation, the supernatant was passed through a 10000 MW filter (Nanosep 10K, Pall). Then, a 20 μl aliquot of sample was analysed for 5-HT and DA content by HPLC coupled to fluorometric detection as previously described [35]. The results were expressed as fmoles per milligram of fresh tissue.

### Slice preparation and electrophysiological recordings

Coronal slices (250 μm thickness) containing PFC were obtained from P21 to P28 mice. They were incubated for at least 1 h at 33°C in the extracellular solution (ES) containing (in mM): NaCl, 126; NaHCO_3_, 26; glucose, 10; CaCl_2_, 2; KCl, 1.5; KH_2_PO_4_, 1.25; MgCl_2_, 2 (pH 7.4, 310–330 mOsm). ES was bubbled continuously with a mixture of 95% O_2_ and 5% CO_2_. Somatic whole-cell recordings of L5PyNs were performed at 33°C, using borosilicate glass pipettes (of 3–5 MΩ resistance in bath) filled with a solution containing (in mM): K-gluconate, 140; HEPES, 10; ATP, 4; MgCl_2_, 2; GTP, 0.4; EGTA, 0.5 (pH 7.3 adjusted with KOH; 270–290 mOsm). Voltage-clamp recordings were performed using an Axopatch 1D (Axon Instruments), filtered by a low-pass Bessel filter with a cutoff frequency set at 2 kHz, and digitally sampled at 4 kHz. The membrane potential was corrected off-line by −10 mV to account for the junction potential. The firing profile of patched neurons and their membrane resistance were obtained using 1 s depolarizing steps ranging from −100 to 200 pA. Only cells having a resting membrane potential more negative than −55 mV and with an access resistance lower than 25 MΩ were kept for further analysis. The access resistance was compensated off-line in voltage clamp mode and neurons exhibiting more than 10% of the access resistance during the experiment were rejected. Electrical stimulations (1–10 μA, 0.2 ms duration) were delivered in layer 2–3 using 1 MΩ impedance bipolar tungsten electrodes (TST33A10KT; WPI). The intensity of the stimulation corresponded to 50% of the maximum response.

Synaptic response analysis and decomposition of synaptic conductance changes used in these studies were similar to those extensively described in previous papers [17,36–39]. Single stimuli applied at low frequency (0.05 Hz) did *not* induce plasticity.

### Chemicals

All chemicals used for electrophysiology experiments were obtained from Sigma-Aldrich except D-L-AP5 and NBQX (from Ascent Scientific). The D1 Receptor agonist SKF 81297 hydrobromide (6-chloro-2,3,4,5-tetrahydro-1-phenyl-1H-3-benzazepine hydrobromide) and the D1R antagonist SCH 23390 hydrochloride ((R)-(+)-7chloro-8-hydroxy-3-methyl-1-phenyl-2,3,4,5-tetrahydro-1H-3-benzazepine hydrochloride) were from Tocris. SKF81297 and SCH23390 were dissolved in water at a 10 mM concentration for stock solutions. Drugs were bath applied for 15 minutes before a “drug recording”.

### Plasticity induction protocol

Under voltage-clamp, five to ten trials were repeated for each holding potential (0.05 Hz). Control recording was made after 15 min of patch-clamp equilibration at five to seven holding potentials. After this control recording, if requested, drug was added to the external medium and superfused on the slice for 15 min. Then a “drug recording” was performed in the same condition of stimulation as for the control recording.

The High Frequency Stimulation (HFS) protocol was elicited in the prefrontal cortical layer 2–3 with theta-burst stimulation (three trains of 13 bursts applied at 5 Hz frequency, each burst containing four pulses at 100 Hz, for a total duration of 2 min); the L5PyN was under current clamp condition (I = 0). Then, recordings of current responses in L5PyNs were done as described above (frequency of stimulation 0.05 Hz) 15, 30, 45 and 60 min after the beginning of the protocol of HFS in order to compare to the control recording.

### Analysis of the plasticity

After a HFS protocol, responses displayed LTP, LTD or no plasticity. These effects were due to the activation of NMDARs and their blockade with D-L-AP5 prevent the induction of plasticity in L5PyNs [17,36]. In order to sort L5PyNs displaying LTP or LTD, we retained an enhancement or a depression of IntgT higher or lower than 20% of the control IntgT before the HFS protocol as already described elsewhere [40,41]. L5PyNs exhibiting an IntgT change ranging between ± 20% of the control conductance before the HFS protocol were classified as “no plasticity”.

### Recording of the NMDA current in L5PyNs

NMDA responses to electrical stimulation (similar to that for the E-I balance determination) of layer 2–3 were recorded after blockade of GABA A receptors by picrotoxin (100 μM) and of AMPA receptors by NBQX (10 μM) at a holding potential of −40 mV. The NMDA nature of the recorded current was confirmed by its complete blockade when applying NMDA receptor blocker D-L-AP5 (100 μM). At least 5 recordings were stacked and averaged.

### Statistical analyses

Data reported are mean ± the standard error of the mean (S.E.M) of n cells. Statistics were calculated using the InVivoStat software (Mockett Media). Statistical significance was evaluated using one way ANOVA to compare unpaired conditions. Paired samples for IntgT, IntgE, IntgI, % E, % I and NMDA currents between the control condition (before HFS) and a given time after HFS (15, 30, 45 or 60 min) were analysed using the parametrical *t*-test. To analyze the orientation of the plasticity, a two-way ANOVA test was used taking into account two categories of parameters, LTP, LTD or no plasticity and time after HFS (15, 30, 45 or 60 min). Data were considered statistically significant for p ≤ 0.05 (*), p ≤ 0.01 (**) and p ≤ 0.001 (***).

## Results

### Serotonin and dopamine levels are significantly enhanced in 5-HT_1A_R-KO mice

**Fig 1:**
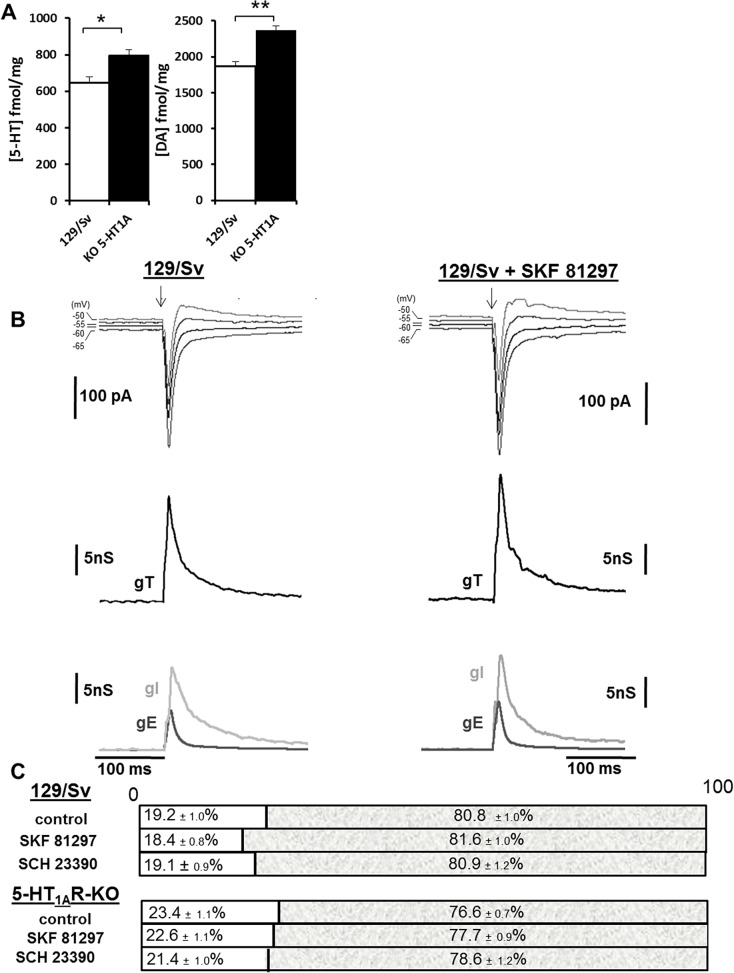
Dopamine does not change the E-I balance in L5PyNs of 129/Sv and 5-HT_1A_R-KO mice. (A) HPLC quantification of 5-HT and DA levels in tissue extracted from PFC of 129/Sv mice (White bar, n = 8, n = 4 animals) and 5-HT1AR-KO (Black bar, n = 8, n = 4 animals). Error bars represent S.E.M. (one-way ANOVA, *p<0.05, ** p< 0.01). (B) First row: Representative current responses in a L5PyN to layer 2/3 stimulation in PFC of a 129/Sv mouse (left column) and in presence of the D1R agonist SKF 81297 (10μM) (right column) recorded under voltage-clamp at various holding potentials (each response is the mean of 5 recordings). Vertical arrows indicate the stimulation onset. Second row: Corresponding conductance changes (gT, black line). Third row: Excitatory (gE, dark grey line) and inhibitory (gI, light grey line) conductance changes obtained from gT decomposition (see Material and Methods). (C) Representation of the E-I balance. The E-I balance does not change significantly in presence of the D1R agonist (SKF 81297, 10μM) or D1R antagonist (SCH 23390, 10μM) whatever the 129/Sv or 5-HT_1A_R-KO mice strain.

It has been shown that 5-HT concentration can modulate the release of DA in the PFC [42]. These effects are likely due to specific localizations of 5-HT1ARs on GABAergic interneurons and on pyramidal neurons in the PFC [42]. A recent study indicates that 5-HT1ARs are required during the critical period (P15-P25) for establishing the circuits that mediate normal anxiety [43]. It is possible that during this critical period that modifies the neuronal circuitry, the lack of 5-HT1ARs induces 5-HT level changes [43]. It was thus of interest to determine the endogenous levels of 5-HT and DA in the PFC tissue extracted from 129/Sv wild type mice or from 5-HT_1A_R-KO mice aged within the critical period for its development. HPLC measurements revealed that 5-HT and DA in mice without 5-HT_1A_R were significantly enhanced by 23.2% (one-way ANOVA, p< 0.05) and 26.7% (one-way ANOVA, p<0.01) respectively (Fig. 1A). Accordingly, the 5-hydroxy indole acetic acid and homovanillic acid metabolites were also enhanced by a similar ratio (data not shown). These results suggest that levels of 5-HT and DA in the PFC are directly or indirectly dependent of the presence of functional 5-HT1ARs. The next step was to better understand the role of 5-HT and DA on plasticity. We first analyzed their effect on the functioning of neuronal networks in the PFC by electrophysiology.

### Effect of D1Rs on the excitation-inhibition (E-I) balance determined in layer 5 pyramidal neurons (L5PyNs) of the PFC

We showed that endogenous DA levels increased in 5-HT1AR-KO mice and this may affect the functioning of neuronal networks. During neuronal activity, the cortical networks are maintained functional through complex interactions between excitatory and inhibitory microcircuits, which permit the expression of a homeostatic regulation of the networks in order to keep the excitation (E)—inhibition (I) balance close to 20–80% excitation-inhibition and to control output signals from the cortex [36]. We showed that the E-I balance, which depends of AMPA and GABA receptors, is modulated by 5-HT [17,38]. In regards of DA, it has been reported that it enhances the amplitude of AMPA currents in the PFC [44] and increases GABAergic interneurons excitability [45]. However, the functional consequences of such complex regulations on the excitatory and inhibitory inputs by DA on L5PyNs excitability have never been investigated.

To study the effect of DA on L5PyNs excitability, we used a technical approach, which allowed us to simultaneously determine the consequences at the somatic level of the modulation of excitation and inhibition inputs. To assess the E-I balance in the medial PFC, we applied an electrical stimulation of layer 2/3 (low frequency stimulation, 0.05 Hz) which evokes a composite (excitatory and inhibitory) postsynaptic current response recorded at various potentials in L5PyNs (Fig. 1B). The total synaptic conductance change (gT) of the evoked response was extracted (see methods) and decomposed into AMPA excitatory conductance (gE) and GABA inhibitory (gI) conductance (Fig. 1B). This allows evaluation of the relative contribution of evoked excitatory and inhibitory inputs reaching the soma of the recorded L5PyN [36,38]. Calculated integrals of excitatory (IntgE) and inhibitory (IntgI) conductances were expressed as percentages of the integral of the total conductance (IntgT) of the response (Fig. 1C).

In a previous study, we evaluated in the PFC the effects of 5-HT1AR deficiency on the E-I balance [17]. The E-I balance was shifted from 20–80% in 129/Sv mice to 23–77% in 5-HT_1A_R-KO mice and this result showed that 5-HT through the activation of postsynaptic 5-HT1ARs modulates the E-I balance [17]. This result was further corroborated by the application of an antagonist of 5-HT_1A_Rs (Way 100635) in 129/Sv wild type mice, which enhanced both excitation and inhibition in different proportion contributing to the E-I balance shifted value similar to the one observed in 5-HT_1A_R-KO mice [17]. In the present study, we first compared the effects of a D1-like receptor agonist, SKF81297 (10 μM) [46] and a D1-like receptor antagonist, SCH23390 (10 μM) [28,47], on 129/Sv wild type mice. We also looked at whether the E-I balance modification observed in 5-HT_1A_R-KO mice [17] could be a consequence of higher levels of DA acting on D1Rs (Fig. 1A).

Stable patch–clamp recordings were obtained from L5PyNs located in the PFC. Recorded neurons from 129/Sv WT (n = 15 cells, n = 8 animals) or from 5-HT_1A_R-KO (n = 15 cells, n = 8 animals) mice had a resting potential of −69.12 ±1.13 mV and −67.32 ± 1.55 mV respectively (no significantly different, one way ANOVA, p = 0.22). Their membrane input resistances were equal to 401.53 ± 43.77 MΩ and 372.77 ± 32.83 MΩ respectively (no significantly different, one way ANOVA, p = 0.35). Bath application of SKF81297 (10 μM) or SCH23390 (10μM) did not change significantly these values.

The effect of DA on the E-I balance was determined in the presence of the D1R agonist, SKF81297 or of the D1R antagonist, SCH23390, on PFC slices of 129/Sv mice and on 5-HT_1A_R-KO mice. We found that D1R agonist enhances both excitation and inhibition in similar proportion (+25%) without modifying the E-I balance value in 129/Sv mice (18.4 ± 0.8%–81.6 ± 1.0%, n = 15 cells, n = 8 animals) or in 5-HT_1A_R-KO mice (22.6 ± 1.1%–77.7 ± 0.9%, n = 15 cells, n = 8 animals). In the case of the D1R antagonist, it had no significant effect on the excitation, the inhibition and the E-I balance in 129/Sv mice (19,2 ±1.0%− 80.8 ±1.0%, n = 15 cells, n = 8 animals) or in 5-HT_1A_R-KO mice (23.4 ±1.1%− 76.6 ± 0.7%, n = 15 cells, n = 8 animals, Fig. 1C). We conclude that D1Rs activation did not modify the E-I balance, whether or not 5-HT_1A_Rs were present on the PFC neuronal network. The next step was then to investigate the role of D1Rs on plasticity.

### Effect of D1Rs activation on LTP-induced in L5PyNs from 129Sv mice and 5-HT1AR-KO mice

Neocortical activities are dominated by fast oscillations in the β/γ range (20–80 Hz) during activated states, whereas the slow oscillation (0.5–1 Hz) is observed during slow-wave sleep [48,49]. In numerous structures of the nervous system and particularly in the cerebral cortex, neuronal activity is often characterized by precise synchronization of discharges [50,51]. Evidence suggests that this synchronization serves response selection in the context of various cognitive functions such as attention, short- and long-term memory [52].

In a previous work in the PFC, we showed that theta burst firing induced either LTP, LTD or no plasticity of responses evoked by layer 2/3 stimulation and recorded in L5PyNs and this distribution of plasticity is modulated towards LTD by 5-HT1ARs activation [17]. DA may facilitate either LTP or LTD depending of basal levels of DA [26,53,54]. Since, we have observed that DA levels increased in 5-HT1AR-KO mice, our aim was to investigate whether DA has the ability to orientate the plasticity through D1Rs activation both in 129/Sv mice and 5-HT1AR-KO mice.

**Fig 2:**
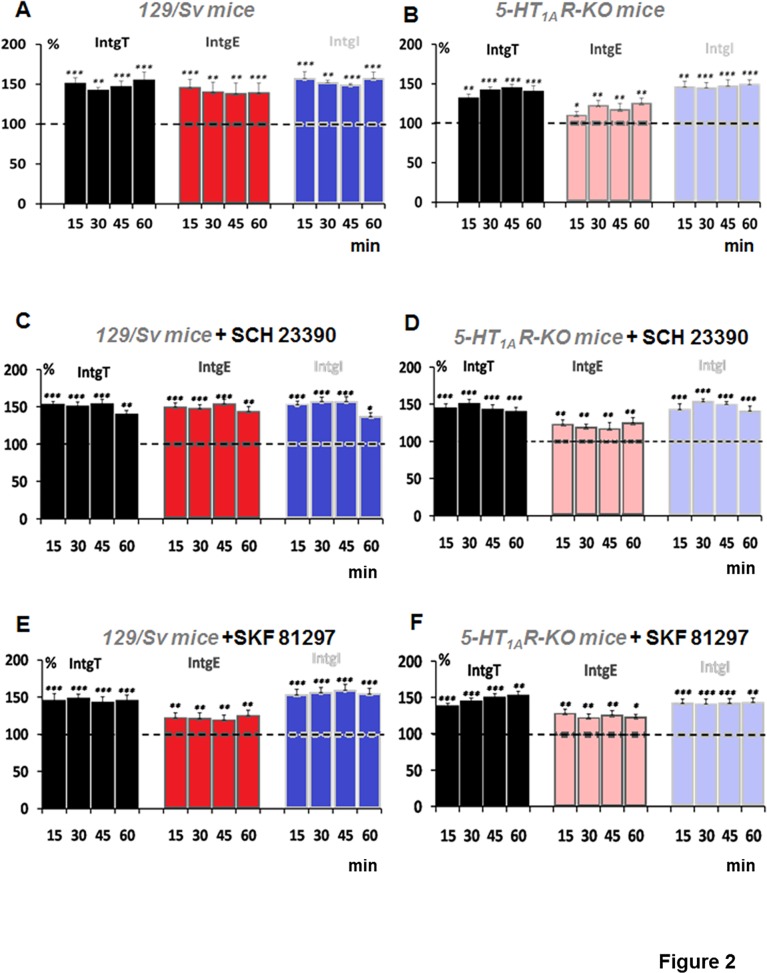
LTP induction by a HFS protocol: D1Rs and 5-HT1ARs modulate the potentiation of the excitation in PFC. (A) LTP responses induced in L5PyNs from 129/Sv mice recorded for 15 min, 30 min, 45 min and 60 min after the HFS protocol. Histograms represent relative changes (compared to control) of conductances, IntgT (black bars), IntgE (red bars) and IntgI (blue bars). Conductances are determined with a low frequency of stimulation (0.05 Hz) in resting conditions or after the HFS protocol. (B) LTP responses induced in L5PyNs from 5-HT_1A_R-KO mice recorded for 15 min, 30 min, 45 min and 60 min after the HFS protocol. (C) LTP responses induced in L5PyNs from 129/Sv mice recorded for 15 min, 30 min, 45 min and 60 min after the HFS protocol in the presence of the D1R antagonist SCH 23390 (10μM). Histograms represent relative conductance changes (compared to the resting condition following 15 min pre-incubation of the antagonist *i*.*e*. 100%), IntgT (black bars), IntgE (red bars) and IntgI (blue bars). (D) LTP responses induced in L5PyNs from 5-HT_1A_R-KO mice in the presence of SCH 23390 (10μM). (E) LTP responses induced in L5PyNs from 129/Sv in the presence of the D1R agonist SKF 81297 (10μM). Histograms represent relative conductance changes (compared to the resting condition following 15 min pre-incubation of the agonist *i*.*e*. 100%), IntgT (black bars), IntgE (red bars) and IntgI (blue bars). (F) LTP responses induced in L5PyNs from 5-HT_1A_R-KO mice in the presence of SKF 81297 (10μM). Error bars represent S.E.M. (* p < 0.05, ** p < 0.01;*** p<0.001).

In the first part of the study, we concentrated our analysis on neurons in which the HFS protocol induced LTP. The Fig. 2A shows potentiation of L5PyNs responses expressed as conductance changes (IntgT, IntgE and IntgI) at various times points (15, 30, 45 and 60 min) after the induction of the HFS protocol (see methods). We found that in average the conductances, IntgT, IntgE and IntgI were enhanced by 49.3 ± 2.7%, 43.8 +/− 2.5% and 54.7 +/− 2.1% respectively in 129/Sv mice (n = 15 cells, n = 8 animals). Importantly, when a HFS protocol was applied on 5-HT1AR-KO mice (n = 15 cells, n = 8 animals), we observed that the potentiation of the excitatory synaptic transmission was significantly weaker than in 129/Sv mice (23.7 +/− 2.5%, p< 0.01) whereas the total synaptic transmission and the inhibitory synaptic transmission were potentiated by 46.7 +/− 2.3% and 48.6% +/− 3.0% respectively (Fig. 2B). These results are in agreement with our previous study [17].

### Effect of D1Rs blockade on HFS-induced LTP in 129/Sv mice and in 5-HT1AR-KO mice

We then investigated the role of D1Rs on HFS-induced LTP of the excitatory and inhibitory transmission. To do so, we blocked D1Rs with the selective antagonist Sch 23390 (10 μM) which was applied 15 minutes before the HFS protocol and remained in the perfusion throughout the experiment. In the presence of the antagonist in 129/Sv mice, the enhancement of conductances (IntgT, IntgE and IntgI) at 15, 30, 45 and 60 min after the HFS protocol was around 50% above the control value (t = 0, Fig. 2C) and similar to those obtained in absence of the antagonist (Fig. 2A).

In 5-HT1AR-KO mice, the potentiation of the excitatory synaptic transmission was significantly weaker than in wild type mice (Fig. 2B). Knowing that DA levels were significantly higher in these mice (Fig. 1A), we next investigated whether DA through activation of D1Rs could play a role in the reduction of the excitatory synaptic transmission observed in 5-HT1AR-KO mice. Our results show that in presence of Sch 23390 (10 μM), in 5-HT1AR-KO mice, the LTP responses were not different from those recorded in absence of Sch 23390 (compare Fig. 2D and 2B).

It appears that the D1R antagonist did not affect the magnitude of LTP in 129/Sv or 5-HT1AR-KO mice.

### Effect of D1Rs activation on LTP-induced in 129/SV mice

We then investigated whether D1Rs activation could modulate the LTP responses. The D1R agonist, SKF 81297 (10 μM) was applied before the HFS protocol and remained in the perfusion throughout the experiment. In the PFC of 129/Sv mice, the excitatory conductance (IntgE) was found only potentiated by 27% versus 43.8% in absence of SKF 81297 (Fig. 2E, 2A) (one way ANOVA, p<0.03) whereas the total and inhibitory conductances (IntgT, IntgI) were potentiated by 46% and 56% respectively and were comparable to the control values.

In 5-HT1AR-KO mice, when a HFS protocol was applied in the presence of SKF 81297, LTP responses were obtained (Fig. 2F). The potentiation of total and inhibitory conductances (IntgT, IntgI) was equal to 47% and 43% respectively, and the excitatory conductance (IntgE) was increased by 25% (Fig. 2F). These potentiating effects were identical to those obtained without the D1R agonist (Fig. 2B). We conclude that the activation of D1Rs selectively reduces HFS-induced potentiation of excitatory inputs (E) in 129/Sv mice but fails to modulate the HFS-induced potentiation of excitation in 5-HT1AR-KO mice. This result suggests that the effect of DA via D1Rs requires functional 5-HT1ARs.

### Effect of D1Rs activation on HFS-induced LTD in L5PyNs from 129/Sv mice and 5-HT1AR-KO mice

**Fig 3:**
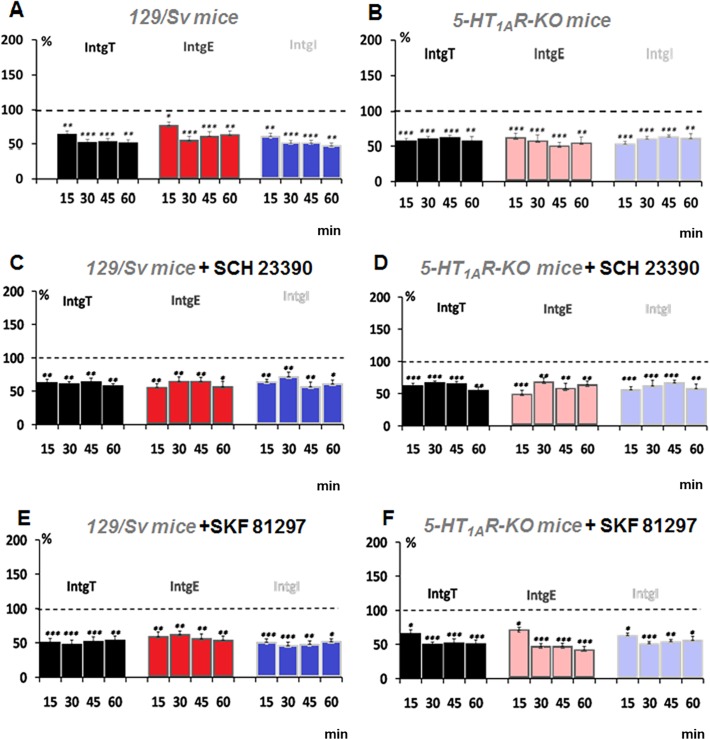
LTD responses induced by a HFS protocol are not modulated by D1Rs and 5-HT1ARs in the PFC. (A) LTD responses induced in L5PyNs from129/Sv mice recorded for 15 min, 30 min, 45 min and 60 min after the HFS protocol. Histograms represent relative conductance changes (compared to resting condition) of IntgT (black bars), IntgE (red bars) and IntgI (blue bars). (B) LTD responses induced in L5PyNs from 5-HT_1A_R-KO mice. (C) LTD responses induced in L5PyNs from 129/Sv mice in the presence of SCH 23390 (10 μM) recorded for 15 min, 30 min, 45 min and 60 min after the HFS protocol. Histograms represent relative conductance changes (compared to the resting condition following 15 min pre-incubation of the antagonist *i*.*e*. 100%), IntgT (black bars), IntgE (red bars) and IntgI (blue bars). (D) LTD responses induced in L5PyNs from 5-HT_1A_R-KO mice in the presence of SCH 23390 (10 μM). (E) LTD responses induced in L5PyNs from 129/Sv mice in the presence of SKF 81297 (10μM). Histograms represent relative conductance changes (compared to the resting condition following 15 min pre-incubation of the agonist *i*.*e*. 100%), IntgT (black bars), IntgE (red bars) and IntgI (blue bars). (F) LTD responses induced in L5PyNs from 5-HT_1A_R-KO mice in the presence of SKF 81297 (10 μM). Error bars represent S.E.M. (* p < 0.05, ** p < 0.01;*** p<0.001).

In the PFC, theta burst firing induced either LTP or LTD responses. Here, we describe the analysis on those neurons, which had displayed LTD. Fig. 3 A and B shows LTD responses obtained in 129Sv mice and 5-HT1AR-KO mice. The conductances, IntgT, IntgE and IntgI were found depressed by 44.1 +/− 2,8%, 38.6 +/− 3.5% and 46.5 +/− 2.7% respectively in 129/Sv mice. In 5-HT1AR-KO mice, conductances were depressed by 51.5 +/− 3.2%, 45.2 +/− 3.4% and by 50.3+/− 3.1% respectively. These results show that the magnitude of the LTD is not changed in 5-HT1AR-KO mice compared with the 129/Sv mice as previously reported [17]. The blockade of D1Rs with Sch 23390 or its activation by SKF 81297 did not modify HFS-induced LTD plasticity (Fig. 3C, D, E, F). Although the magnitude of LTD was not changed, the analysis of our results on plasticity in L5PyNs indicates that the probability of the L5PyNs population to display LTP, LTD or no plasticity has changed.

### Orientation of the plasticity by 5-HT1ARs and D1Rs

**Fig 4:**
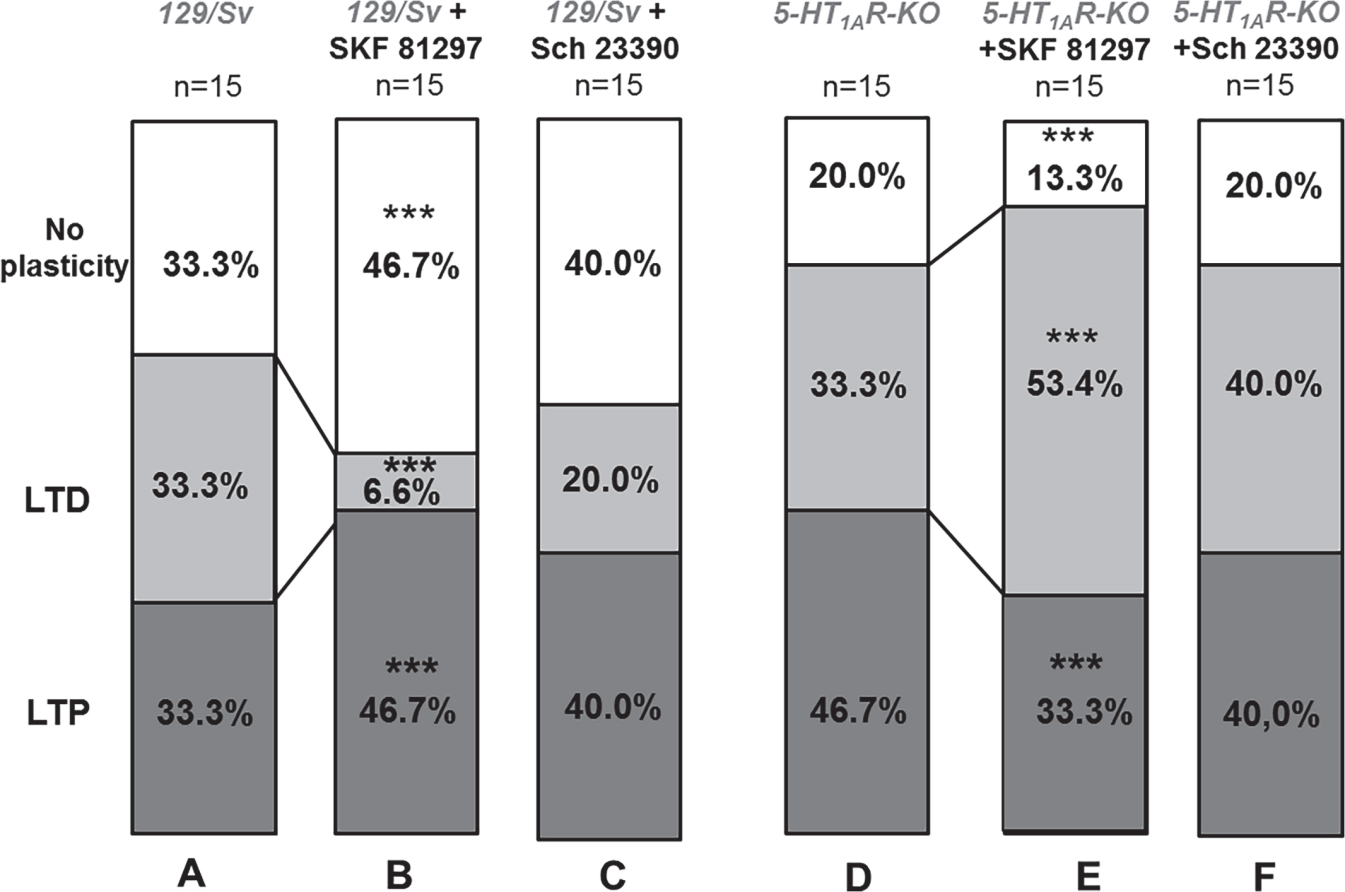
Changes after 45 minutes in the proportion of LTP, LTD or no plasticity responses observed in L5PyNs population induced by the HFS protocol in the presence of D1R agonist or antagonist in 129/Sv and 5-HT_1A_R-KO mutants (expressed as percentages, n = 15 for each condition). (A and D) Percentages of L5PyNs displaying LTP, LTD or no plasticity in 129/Sv mice and 5-HT_1A_R-KO mice in control conditions. (B) In 129/Sv mice, the D1R agonist SKF 81297 enhanced the percentage of L5PyNs displaying LTP and “no plasticity” while the percentage of L5PyNs displaying LTD was significantly decreased (two-way ANOVA, p < 0.001). (C) In 129/Sv mice, the D1R antagonist SKF 23390 did not change significantly the percentages of L5PyNs displaying LTP, LTD or “no plasticity” (compare columns A and C). (E) In 5-HT_1A_R-KO mice, the D1R agonist SKF 81297 significantly increased the percentage of L5PyNs displaying LTD and significantly reduced the LTP and the “no plasticity” groups (two-way ANOVA, p < 0.001). (F) In 5-HT_1A_R-KO mice, the D1R antagonist SKF 23390 did not change significantly the percentages of L5PyNs displaying LTP, LTD or “no plasticity” (compare columns D and F). (* p < 0.05, ** p < 0.01;*** p<0.001).

We analyzed the whole population of recorded L5PyNs, to extract the percentage of L5PyNs showing LTP, LTD or no plasticity following a HFS protocol in the control condition, in the presence of a D1R blocker (SCH 23390), or D1R activator (SKF 81297). In 129/Sv mice, the percentages of L5PyNs displaying LTP, LTD or “no plasticity” in the presence of the D1R blocker (Fig. 4C, n = 15) were 40.0%, 20.0% and 40.0% respectively. We found that in 5-HT1AR-KO mice, the percentage of L5PyNs displaying LTP, LTD or no plasticity has changed to more neurons displaying LTP (46.7%) and less neurons showing ‘no plasticity” (20.0%) (two-way ANOVA, p<0.001) compare to the control condition (Fig. 4D, A). In our experimental conditions both in 129/Sv and in 5-HT1AR-KO mice, the D1R blocker had not effect on the proportion of L5PyNs population displaying LTP, LTD or no plasticity (Fig. 4C, F). On the contrary, the activation of D1Rs in 129/Sv mice induced (Fig. 4A, B) an increase of L5PyNs displaying LTP (46.7% of the population) and a strong reduction of L5PyNs displaying LTD (6.6% of the population) whereas the “no plasticity” group was slightly increased (46.7% of the population) (two-way ANOVA, p<0.001). Surprisingly, in 5-HT1AR-KO mice (Fig. 4D, E), the activation of D1Rs resulted in an increase of L5PyNs displaying LTD from 33.3% to 53.4% of the population (two-way ANOVA, p<0.001), accompanied by a marked decrease of the “no plasticity” group (from 20.0% to 13.3%, two-way ANOVA, p<0.001) and a reduction of the proportion of L5PyNs displaying LTP from 46.7% to 33.3% (Fig. 4D, E).

Finally, it appears that both 5-HT1ARs and D1Rs are essential to fine tune the plasticity of L5PyNs. D1Rs activation increases the LTP in 129/Sv mice. In contrast, application of D1Rs agonist on neurons lacking the 5-HT1AR facilitates LTD and decreases LTP and “no plasticity” groups. Our results suggest that the effect of D1R agonist on plasticity depend of the presence of 5-HT1ARs.

### Modulation of the NMDA current by D1Rs in 129/Sv mice and 5-HT1AR-KO mice

Although D1Rs appeared important for the fine tuning of plasticity in L5PyNs, the analysis of plasticity in single L5PyNs (Fig. 2) only revealed a moderate decrease of the potentiation of excitation (AMPA-dependent) after the HFS protocol. However, it is well documented that NMDARs (activated by glutamate during the HFS protocol) is central to direct synaptic plasticity since the induction of plasticity partly relies on the NMDA-dependent Ca^2+^ influx assuming that a small or moderate postsynaptic concentration of Ca^2+^ leads to LTD whereas a large activation of NMDARs causes LTP [55–58]. To further understand our results, it was then of interest to analyze the effects of D1Rs activation on NMDA currents in L5PyNs. The hypothesis is that the regulation of NMDARs could provide the mechanisms underlying the orientation of the plasticity observed in our experimental condition. D1Rs activation has been shown to powerfully modulate NMDA currents whereas these currents are moderately enhanced in the mouse PFC by the activation of 5-HT1ARs [17,24].

**Fig 5:**
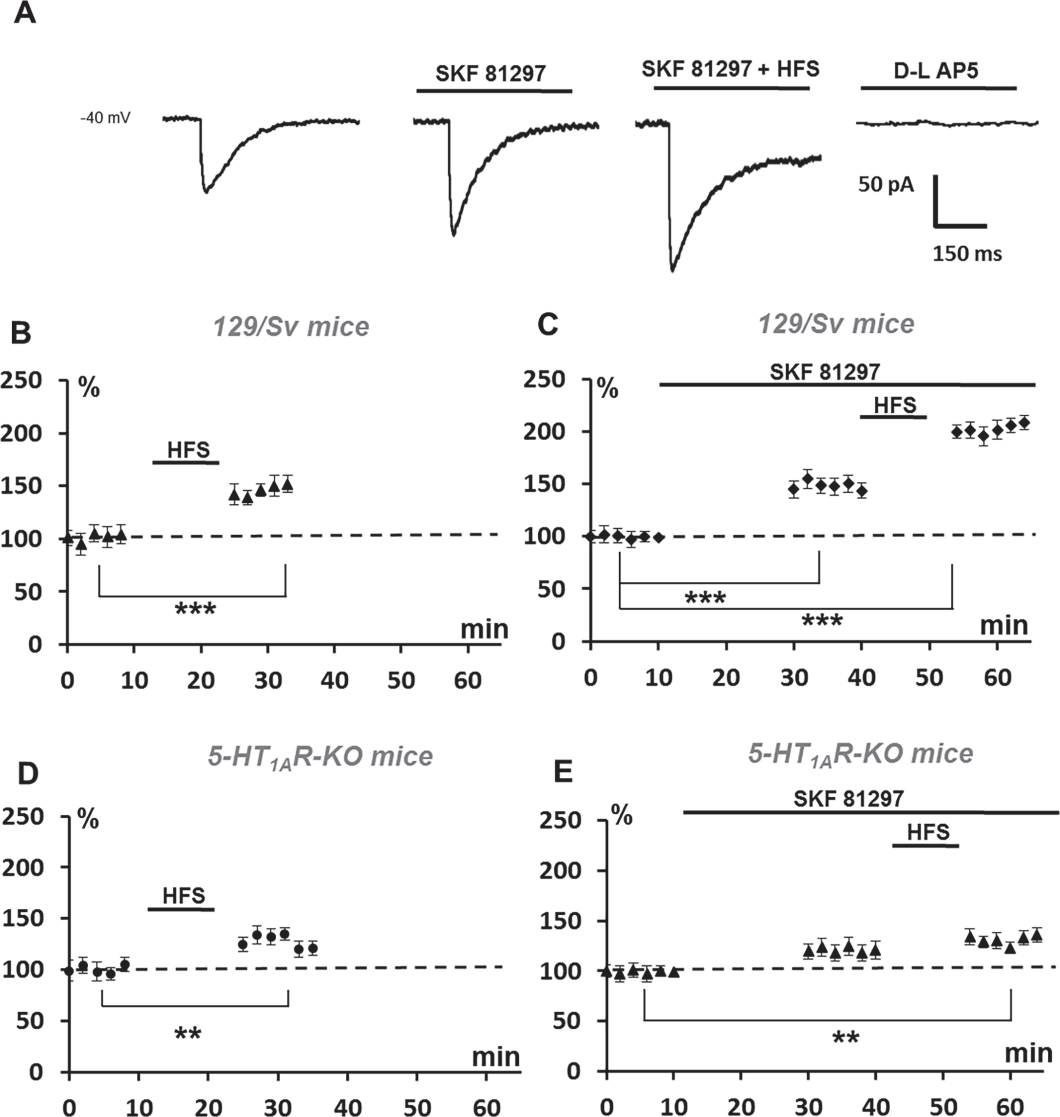
Modulatory effect of 5-HT_1A_Rs and D1Rs on NMDA current recorded in a L5PyN. (A) Typical recordings of the NMDA current in the same L5PyN from a 129/Sv mouse (see method). The amplitude of the current was markedly increased by SKF 81297 (10μM) and was further increased by the application of the HFS protocol in layer 2–3. The current was completely blocked by D-L-AP5 (100 μM), the selective blocker of NMDA receptors. (B) In 129/Sv mice, the normalized NMDA current amplitude was increased by 43.2 +/− 2.1% (n = 5 cells) after the HFS protocol. (C) In 129/Sv mice, the SKF 81297 (10μM) increased the NMDA current amplitude by 48.6+/− 2.5% (n = 5 cells). After the HFS protocol, the NMDA current was further increased to 197.5 +/− 3.1% of the normalized control value (p < 0.001). (D) In 5-HT_1A_R-KO mice, the normalized NMDA current amplitude was increased by 24.2 +/− 1.1% (n = 5 cells) after the HFS protocol. (E) In 5-HT_1A_R-KO mice, the SKF 81297 (10μM) enhanced the NMDA current amplitude by 21.4 +/− 3.1%. After the HFS protocol, the NMDA current was increased to 33.6 +/− 2.5% of the normalized control value (p < 0.01). Error bars represent S.E.M. (* p < 0.05, ** p < 0.01;*** p<0.001).

In L5PyNs of 129/Sv mice, the inward NMDA current evoked by a stimulation in layer 2–3, was increased by 48.6 +/− 2.5% in the presence of the D1R agonist SKF 81197 (10 μM) and was noticeably further enhanced after a HFS protocol to 197.5 +/− 3.1% (one way ANOVA, p<0.001, n = 5 cells, n = 3 animals) (Fig. 5A, C). In 5-HT1AR-KO mice, SKF 81197 only enhanced the NMDA current by 21.4 +/− 3.1% (Fig. 5E) and then the HFS protocol did not enhance the NMDA current by more than 33.6 +/− 2.5% (Fig. 5E) of the control level (one way ANOVA, p<0.01, n = 5 cells, n = 3 animals). The lack of 5-HT1ARs markedly reduces the increase of the NMDA current normally observed in the presence of the D1 agonist in 129/Sv mice. It appears that NMDA currents are either strongly or weakly modulated by the activation of D1Rs depending of the presence of 5-HT1ARs and this may underlie the orientation of plasticity.

## Discussion

DA and 5-HT coexist in the PFC [59,60] and 5-HT1ARs are involved in the modulation of dopaminergic activity [42]. In this work, we show that D1Rs activation orientates plasticity towards LTP and that a lack of 5-HT1ARs limits the increase of the NMDA current normally observed in the presence of the D1 agonist. We also observed that the plasticity in 5-HT1AR-KO mice is orientated towards a large percentage of L5PyNs displaying LTD. Our results reveal a prominent role of 5-HT1ARs in dopamine-induced modulation of plasticity.

### Modulation of the E-I balance

The D1R agonist SKF 81297 has been shown to enhance the amplitude of evoked EPSCs via an increase of AMPA receptors recruitment in the PFC [44]. Furthermore, SKF 81297 is known to increase interneuron excitability that leads to enhanced release of GABA with a delay [45]. Both observations support our finding that the E-I balance is not modified in the presence of this D1R agonist since the AMPA-dependent excitation (E) and the GABAergic inhibition (I) are increased by identical proportions. In contrast, it has been shown that the D1R antagonist Sch 23390 did not enhance EPSCs and IPSCs recorded in pyramidal neurons of PFC [44,45]. In our experiments, we reinforce these previous observations since Sch 23390 did not change the excitation and inhibition thus maintaining the E-I balance stable.

### Plasticity of excitation and inhibition

Although the value of the E-I balance appears not altered, the situation is different when a HFS protocol is applied to induce plasticity. In L5PyNs displaying LTP, the AMPA-dependent excitation (E) was increased to a lesser extent than the GABAergic dependent inhibition (I) following the HFS protocol when a D1R agonist was applied in 129/Sv mice. It is striking that this mild increase of excitation is not associated with an increase of inhibition by the same proportion as expected by homeostatic plasticity processes [36]. One possible explanation, whatever the mouse strain, is the localization of D1Rs on GABAergic interneurons which increases their excitability [23] and in turn enhances the inhibitory effect on pyramidal neurons *i*.*e*. the inhibition I [61]. Both D1Rs activation and HFS-induced LTP of excitation evoke phosphorylation of AMPARs and/or mobilization of new AMPARs at the synapses through lateral diffusion of AMPARs and AMPARs exocytosis [62,63]. Our data on the E-I balance indicates that application of SKF 81297 increases AMPAR currents, which may reduce thereby the number of mobilisable AMPARs by the HFS protocol. Finally, we could not ruled out that since D1Rs activation strongly enhance the NMDA current (Fig. 5B), this could lead to a major Ca^2+^ influx that may interfere with AMPA trafficking by stimulating for instance the endocytosis of AMPARs and thereby reducing the extent of the LTP magnitude. In support of this possibility, it has been reported in hippocampus that the activation of NMDARs triggers the endocytosis of AMPARs [64,65].

### Orientation of the plasticity of L5PyNs in the PFC

An important characteristic of the PFC, is that the HFS protocol induces either LTP, LTD or no plasticity. It is well known that DA could favor LTP induction as observed for instance at hippocampal-PFC synapses [24,25]. Our result showed that following D1Rs activation, the HFS protocol led to an increase of the population of L5PyNs displaying LTP in 129/Sv mice. This result is in agreement with the fact that DA through D1Rs activation enhances NMDA currents in L5PyNs as observed in our study and by others [24,66]. We also observed in 129/Sv mice, an increase in the number of L5PyNs in the “no plasticity” class after D1Rs activation. Since we have observed that application of SKF 81297 by itself increases the AMPA-dependent excitation (E), the simplest explanation is that some L5PyNs became insensitive to a HFS protocol therefore contributing to the “no plasticity” class. Finally, a strong increase of the Ca^2+^ concentration due to NMDARs activation in dendritic spines of L5PyNs may lead to either facilitation of LTP-induced plasticity (Fig. 4) or an absence of the induction of plasticity. A possibility to explain our data is that the magnitude of Ca^2+^ entry through the NMDA receptor could lead to different states of CamKII activation or inhibition as reported in other studies [67,68,69] and consequently could lead to LTP, LTD or “no plasticity”.

In our experiments with 5-HT1AR-KO mice compared with 129/Sv mice, we observed an increase of the population of L5PyNs displaying LTP with a reduction of the “no plasticity” population. In our previous study, we have shown that application of an antagonist of 5-HT1AR mimics the effect of 5-HT1AR-KO mice on the orientation of plasticity, indicating that the plasticity was not affected by developmental remodeling [17].

When we applied a HFS protocol in presence of the D1 agonist on neurons lacking the 5-HT1AR, the population of L5PyNs displaying “LTD” was even more increased at the expense of neurons displaying either LTP or “no plasticity”. It is surprising that D1Rs activation mainly increase the LTD group contrary to what we have observed in 129/Sv. One possibility is that adaptive changes in D1, D2 and NMDA receptors functionality in 5-HT1AR-KO mice have occurred [70]. D1Rs and D2Rs activation are known to modulate NMDARs depending of NMDA subunits (NR2B vs NR2A) [71,72], which are crucial in orientating synaptic plasticity because intracellular Ca^2+^ signaling leads to either LTD or LTP according to the amplitude and the duration of the Ca^2+^ transient [56,58]. The large proportion of neurons showing LTD responses in 5-HT1AR-KO mice could result from the weak enhancement of the NMDA current (Fig. 5) in the presence of D1R agonist which may reflect an adaptive change in subunits composition of NMDARs when 5-HT1ARs are lacking and this effect could favor LTD induction.

It is striking that in our experiments in 129/Sv mice, activation of D1Rs impaired LTD induction. This result paralleled a recent study where a high level of DA has been reported in the rat PFC to impair LTD induction through D1Rs activation [28]. The specific D1Rs activation in these experimental conditions is corroborated by many other observations [28,73] and supported by the high affinity of D1Rs for DA (National Institute of Mental Health Psychoactive Drug Screening Program database), however the mechanism involved in the impairment of the LTD induction is not yet elucidated. It is well known that D1Rs activation recruits G_s_-dependent cAMP pathway that lead to PKA-dependent phosphorylation of AMPAR and thereby modulating synaptic plasticity [24,74,75]. In the case of 5-HT1ARs, the cAMP/PKA pathway is the main signaling pathway recruited [76]. Our data show that D1Rs activation requires functional 5-HT1ARs to be efficient on plasticity, i.e. to increase the number of L5PyNs displaying LTP and decrease the number of L5PyNs displaying LTD. One possibility to explain such 5-HT1ARs requirement for D1R-dependent LTP modulation is that both of these metabotropic receptors are link to Gs and both activate the PKA-dependent pathway. In absence of 5-HT1ARs and in presence of D1Rs agonist, a HFS protocol induced an increase in the number of L5PyNs displaying LTD. In this case, one possible explanation is that the D1Rs activation would not be able to fully activate the Gs-dependent PKA pathway limiting thereby the AMPARs recruitment. There are also growing evidence, including in the PFC, that D1/D5R stimulation lead to Gq-dependent PLC activation, IP_3_-evoked Ca^2+^ release and CamKII activation [67]. It is important to note that several studies have reported that PLC-dependent metabotropic receptor stimulation lead to LTD induction [77]. In the 5-HT1ARs KO mice, the D1Rs effects could mostly rely on the PLC pathway activation, which might explain the LTD induction observed in our study.

### Functional implication

A recent study in the PFC shows that the joint activation of DA and 5-HT systems regulates entering information, local activity and so participates in the modulation of the neuronal activity [29]. Indeed any dysfunction of DA and/or 5-HT receptors will disrupt not only cognitive functions of the PFC but also the functions of subcortical nuclei. In schizophrenia, a pathological conversion of LTP to LTD occurs in the PFC when the basal release of DA is low and NMDAR efficiency is restrained [13]. Our results could bring new evidence for the molecular basis of such phenomenon. We observed a modulation of the dynamics determining the polarity of LTP/LTD-like changes depending of the interaction of D1Rs and the presence or not of 5-HT1ARs in link with NMDA current. In pathological situation such as depression where 5-HT1ARs expression varies, dopaminergic treatments used for their ability to increase LTP could turn in fact to be less and less effective.
